# Clinical and Economic Benefits of Autologous Epidermal Grafting

**DOI:** 10.7759/cureus.875

**Published:** 2016-11-11

**Authors:** Andrea D Maderal, Robert S Kirsner

**Affiliations:** 1 Department of Dermatology and Cutaneous Surgery, University of Miami, Miller School of Medicine

**Keywords:** chronic wounds, epidermal grafting, venous ulcers

## Abstract

Chronic wounds are an increasingly prevalent disease with a significant healthcare burden. These wounds often do not respond to standard of care therapy alone, requiring the use of adjuvant therapies. Epidermal grafting, previously utilized primarily for correction of leukoderma, is increasingly being recognized as a beneficial therapy for wounds, both acute and chronic. Epidermal grafting has been shown to be effective in the management of chronic wounds, with successful healing in refractory patients. It has not only been shown to be effective, but it is also associated with lower cost and morbidity than traditional skin grafting techniques as well as improved donor site healing. Through the use of a novel epidermal harvesting system, the CelluTome™ Epidermal Harvesting System (KCI, an Acelity company, San Antonio, TX), this treatment modality has become more standardized, reproducible, and easy to use as well as less time consuming, making its use in the clinical setting more convenient and beneficial. Epidermal grafting, therefore, represents a promising, efficacious, and cost-effective option for treatment of refractory non-healing wounds.

## Introduction

Chronic wounds are common, difficult to treat and expensive. As an example, venous leg ulcers (VLU) affect over two million Americans, cost $15 billion annually and depending on their size and duration, only 30-75% of patients heal after six months of standard care [1]. For difficult-to-heal ulcers, skin grafting techniques are often utilized to assist in wound healing. Various forms of skin grafting exist and are traditionally divided into full-thickness skin grafts (FTSG), which consist of grafts containing the epidermis and the entire dermis; split-thickness skin grafts (STSG), which consist of grafts containing the epidermis and part of the dermis; and epidermal grafts, which consist of grafts containing the epidermal tissue alone [2]. FTSG are harvested by surgical excision while the donor site is treated with primary closure. This modality is only possible for select areas where there is sufficient skin laxity, and hence limits the applicability of use [2]. STSG, on the other hand, are usually harvested by use of a dermatome which can shave a portion of skin, the depth of which can be adjusted by settings of the dermatome, leaving the deeper, reticular dermis intact. This method creates a wound at the donor site that carries risk of infection, scarring and poor wound healing [2]. FTSG are commonly used to prevent wound contraction when cosmesis is an important consideration, such as on the face. STSG are traditionally used in the treatment of chronic lower extremity wounds as their thinner nature requires less vascular supply and there is increased graft success [3]. In some instances, newer therapies, such as cellular and tissue-based products, have been replacing the use of STSG due to the limitations imparted by the high-risk nature of these procedures (need for creation of donor site, costs, etc.). In many cases, STSG and FTSG require hospitalization and/or the use of a surgical operating room, administration of anesthesia and in some cases, periods of immobility, which limit their use [2,4]. Epidermal grafting is a method that allows autologous skin grafting in an outpatient setting, minimizing the costs and morbidities associated with STSG and FTSG [2].

First introduced by Kiistala and Mustakallio in 1964, epidermal skin grafting has traditionally been performed by the suction blister technique [5]. This technique results in cleavage through the lamina lucida as it is the weakest part of the skin and subject to cleavage with suction. This preserves the ultrastructure of the epidermis with only patchy hemidesmosome disruption [6]. Traditionally, it has been used in dermatology for treatment of leukoderma [7] but has been shown to be effective in the management of acute surgical wounds and chronic ulcers [8]. Its use in the treatment of chronic wounds has been limited to date because of the previously tedious and time-consuming processes to produce suction blisters [8]. A new, commercially available, automated system for epidermal harvesting, the CELLUTOME Epidermal Harvesting System, minimizes these factors; it has been shown to be less time-consuming and more standardized and reproducible than former methods [4]. It functions by applying heat and suction concurrently to normal skin at the donor site to induce small blisters, or microdomes, within 30-45 minutes [9]. These epidermal samples are then transferred via transparent film (or other adhesive) dressing to the recipient sites, which are then wrapped in compression. Suturing or other procedures are not necessary, as they are for STSG and FTSG.

Epidermal grafting has several benefits over STSG and FTSG. Firstly, the donor site experiences less pain and scarring as compared to other modalities. The epidermis lacks pain sensory nerves; therefore, patients experience minimal discomfort and do not require anesthesia for the procedure [4]. The donor site also heals rapidly, within several days, with very minimal and most commonly, no scarring, as the dermis is left intact [10]. Secondly, epidermal grafts are suitable for lower extremity and less vascularized wounds, as the thinner graft requires less vasculature to remain viable [10]. Thirdly, the procedure is simplified, automated and does not require surgical training, increasing ease of use [4]. Finally, in many instances the procedure is more cost-effective than STSG and FTSG as it can be performed on an outpatient basis or at the bedside.

Epidermal grafting has been reported to be beneficial in treatment of various patients including those with acute wounds [11], chronic ischemic wounds [10], chronic diabetic foot ulcers [12], and ulcers in patients with autoimmune connective tissue disease [13]. Epidermal grafting was found to be a successful treatment in a case series of five patients with pyoderma gangrenosum, where the risk of pathergy often limits treatment with other forms of grafting [14]. Pathergy is a phenomenon whereby trauma may lead to worsening of the wound, and creation of a donor site, as would be needed for STSG, would be a contraindication. In this series, all five patients experienced substantial wound size reduction, and three out of five experienced wound closure. Importantly, all donor sites healed without sequelae [14].

Epidermal grafting using cultured keratinocyte sheets has also been compared to STSG in the treatment of recalcitrant vascular leg ulcers [15]. In a multicenter, randomized phase II trial by Tausche et al., 91 subjects with chronic leg ulcers were treated either with traditional STSG or a tissue-engineered, autologous epidermis equivalent. At 12 weeks, the two groups were comparable, with a 30% closure rate in the STSG group and a 31% closure rate in the epidermal grafting group [15]. But at six months, a difference was noted. There was a 34% closure rate in the STSG group and 44% in the epidermal grafting group, demonstrating that only the epidermal grafting group continued to heal after six weeks, whereas the STSG group did not.

For the cases presented in this article, informed consent was obtained from the patients for treatment.

## Materials and methods

**Figure 1:**
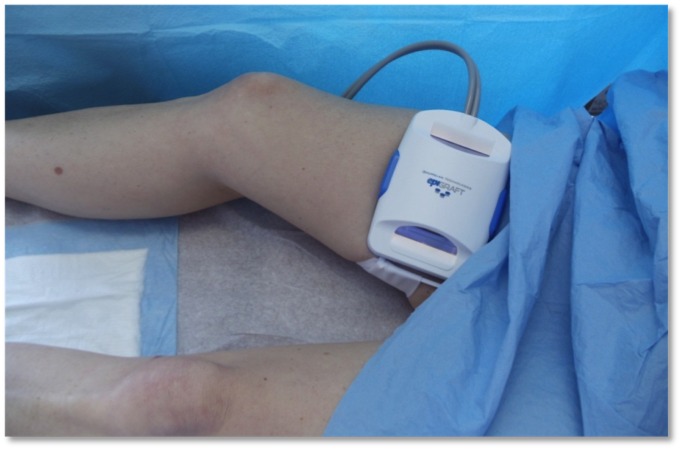
CELLUTOME Epidermal Harvesting System

A new, automated system for epidermal harvesting, the CELLUTOME Epidermal Harvesting System, has been utilized in our clinic for the treatment of chronic wounds. The system consists of a control unit, vacuum head, and harvester that is applied to intact skin on the thigh (Figure 1).

This system simultaneously warms the skin at a temperature of 40 degrees Celsius and administers a negative pressure of 200 mmHg to harvest. Both a smaller harvester that creates 42 epidermal microdomes, covering a 2.5 x 1.75 cm area, and a larger harvester that creates 128 epidermal microdomes are available [14]. The harvesting time ranges from 15 to 60 minutes, on average lasting 30 minutes. The epidermal grafts are then transferred to the recipient site via transparent film dressing and secured in place with compression wraps, bolster dressing, or any other dressing indicated by the clinician. The donor site is simply covered with a transparent film dressing.

Through this technique, these small microdomes are cleaved consistently through the lamina lucida, as confirmed by histologic examination [16]. This method retains the original structure of the keratinocytes and maintains the proliferative activity of the basal keratinocytes that secrete various factors important for wound healing, including vascular endothelial growth factor (VEGF), transforming growth factor-alpha (TGF-a), platelet-derived growth factor (PDGF), hepatocyte growth factor (HGF), and granulocyte colony stimulating factor (G-CSF) [16]. A preclinical trial performed on 12 healthy volunteers demonstrated that using this technique, on average, 99.5% of the epidermal microdomes remained viable after harvesting [17].

The CELLUTOME Epidermal Harvesting System has also been evaluated in small patient populations. In one pilot study of 35 patients with acute and chronic wounds, 22 patients (62.9%) achieved complete wound closure [18]. The mean pain score during graft harvest was 1.42 and the donor site Vancouver Scar Scale was zero for all cases at six weeks, with a mean time to donor site healing of 5.49 days. In another patient series by Gabriel et al. of four patients treated with this epidermal harvesting system, there was complete wound closure in three out of four wounds and 50% closure in the other, and donor sites healed completely without scarring within one to two weeks [9]. Currently, this system is being studied in a randomized, controlled, parallel-group, multicenter study to investigate the efficacy of epidermal grafting against STSG [19].

The CELLUTOME Epidermal Harvesting System has also been used successfully on several patients with chronic wounds at the University of Miami, including wounds due to venous insufficiency, pyoderma gangrenosum, sickle cell disease, post-surgical wounds, and traumatic wounds. These cases are discussed below.

## Results

### Clinical improvement in wound healing

Case One

**Figure 2:**
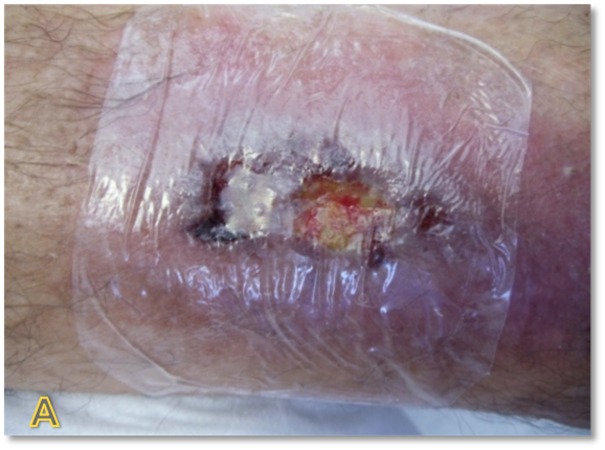
Case One - Day Zero

**Figure 3:**
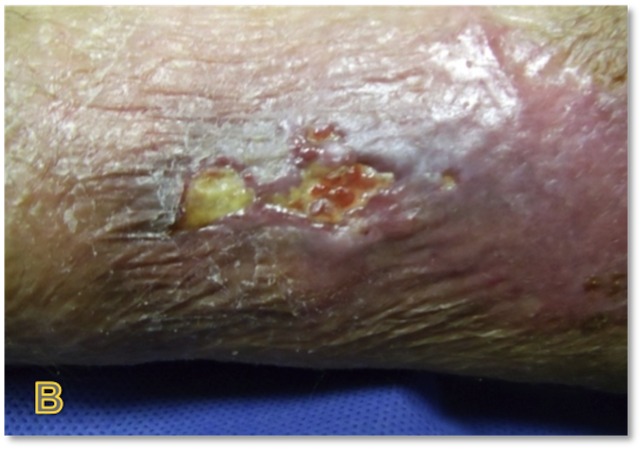
Case One - Day Eight

**Figure 4:**
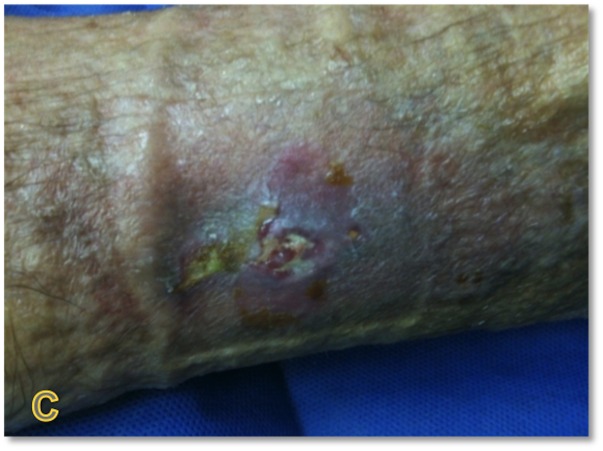
Case One - Day 21

**Figure 5:**
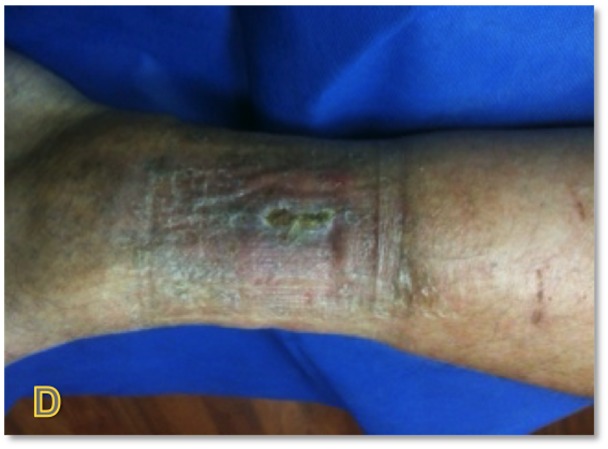
Case One - Day 35

**Figure 6:**
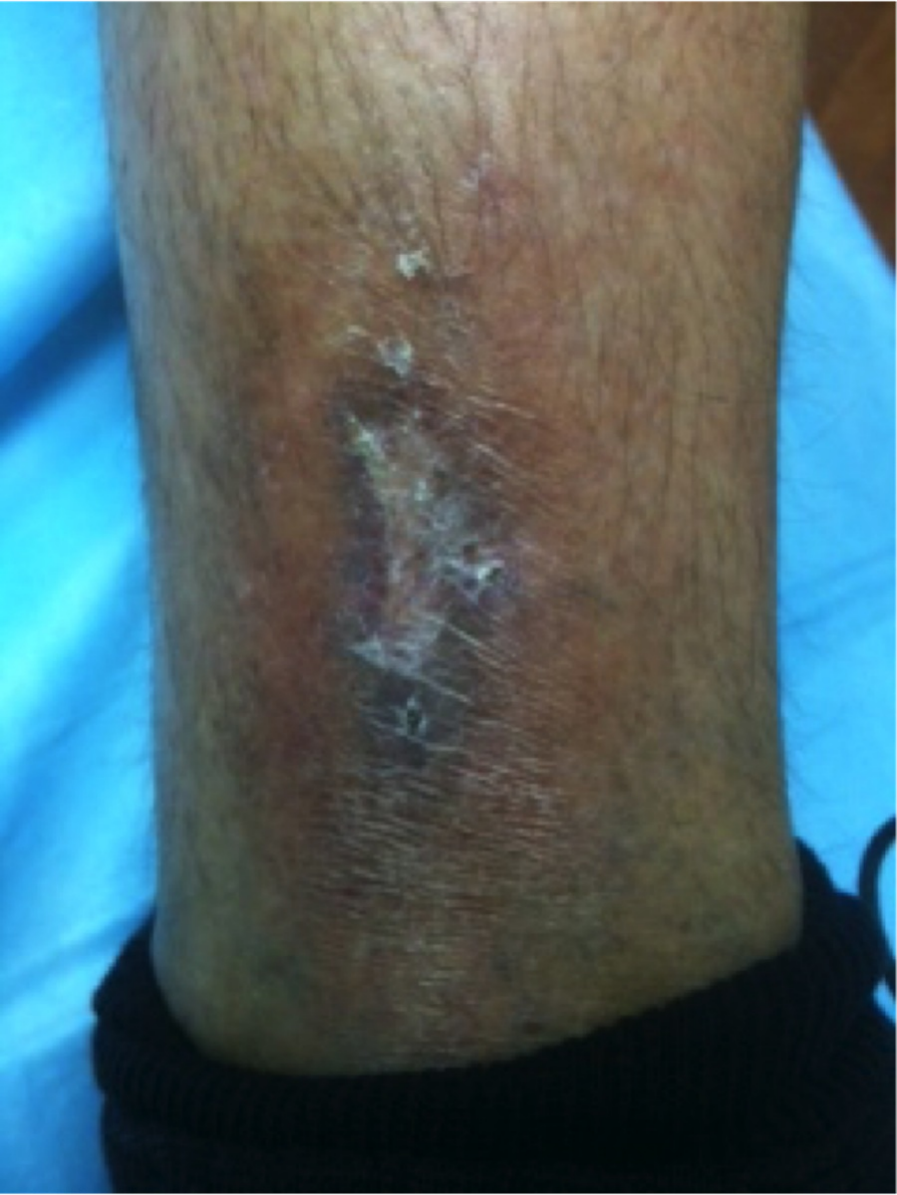
Case One - Day 60

The patient is a 45-year-old male with venous insufficiency and pyoderma gangrenosum who presented to the clinic with a non-healing leg ulcer for six months. The patient was currently managed on systemic steroids and previously had failed application of a bilayered living skin equivalent. Epidermal harvesting was performed using the CELLUTOME Epidermal Harvesting System from intact skin on the right thigh and transferred to the recipient site on the right lateral leg via transparent film dressing (Figure 2). The patient was followed sequentially with improvement in size of wound at day eight, day 21, and day 35, until complete wound closure was achieved at day 60 (Figures 3-6).

Case Two

**Figure 7:**
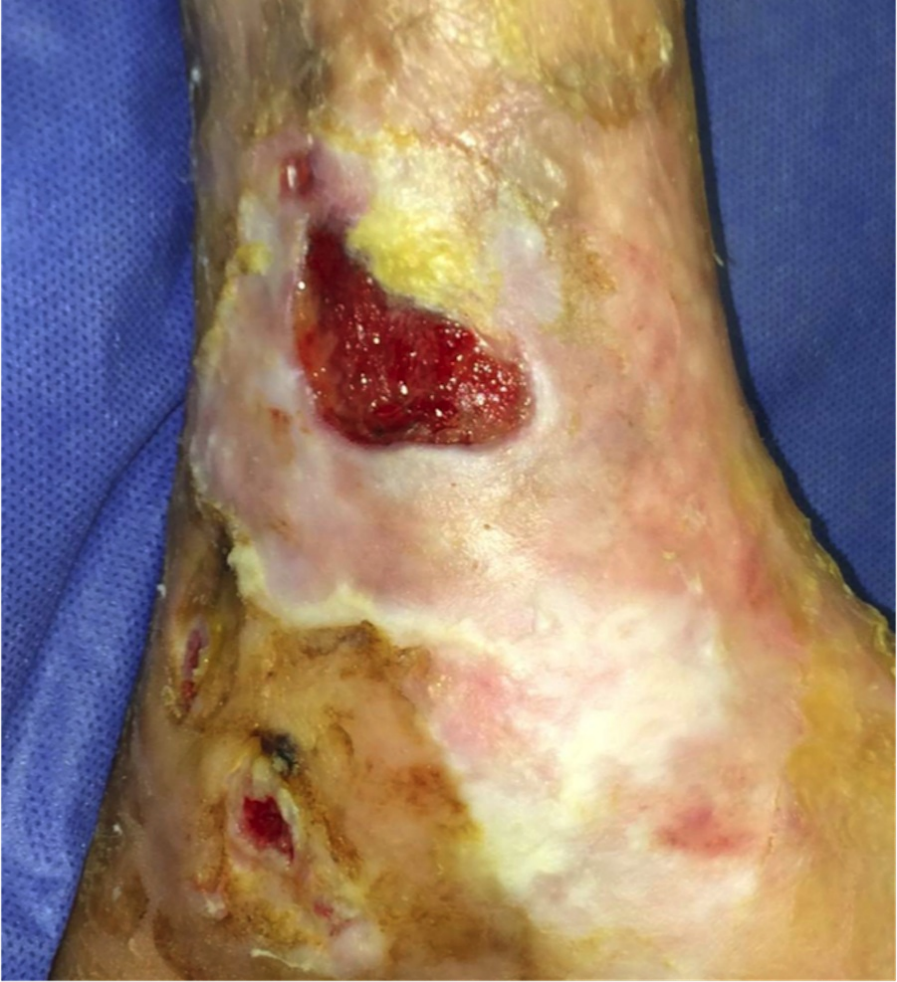
Case Two - Day Zero

**Figure 8:**
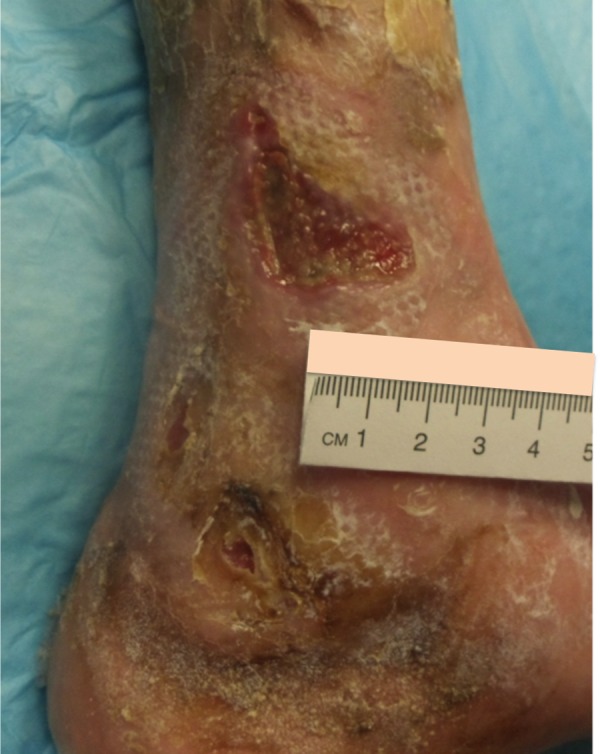
Case Two - Day 20

The patient is a 78-year-old female with an 18-month history of non-healing ulcer on the leg due to venous insufficiency. Past treatments included bilayered living skin equivalent, small intestine submucosa, and compression therapy. Epidermal harvesting was performed using the CELLUTOME Epidermal Harvesting System from intact skin on the right thigh and transferred to the recipient site on the left medial leg via transparent film dressing. At day 20, the patient had significant reduction in size and depth of the ulcers as compared to pre-grafting (Figures 7-8).

Case Three

**Figure 9:**
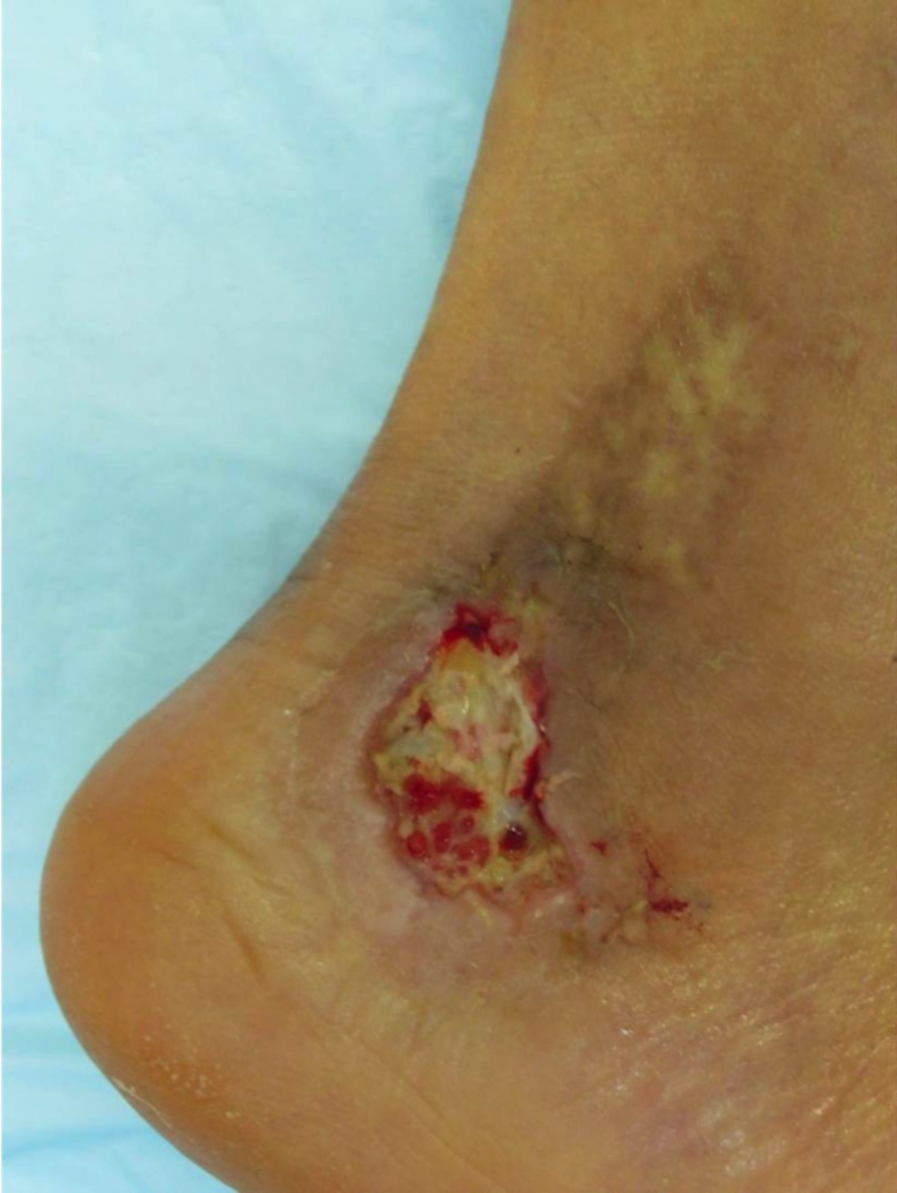
Case Three - Day Zero

**Figure 10:**
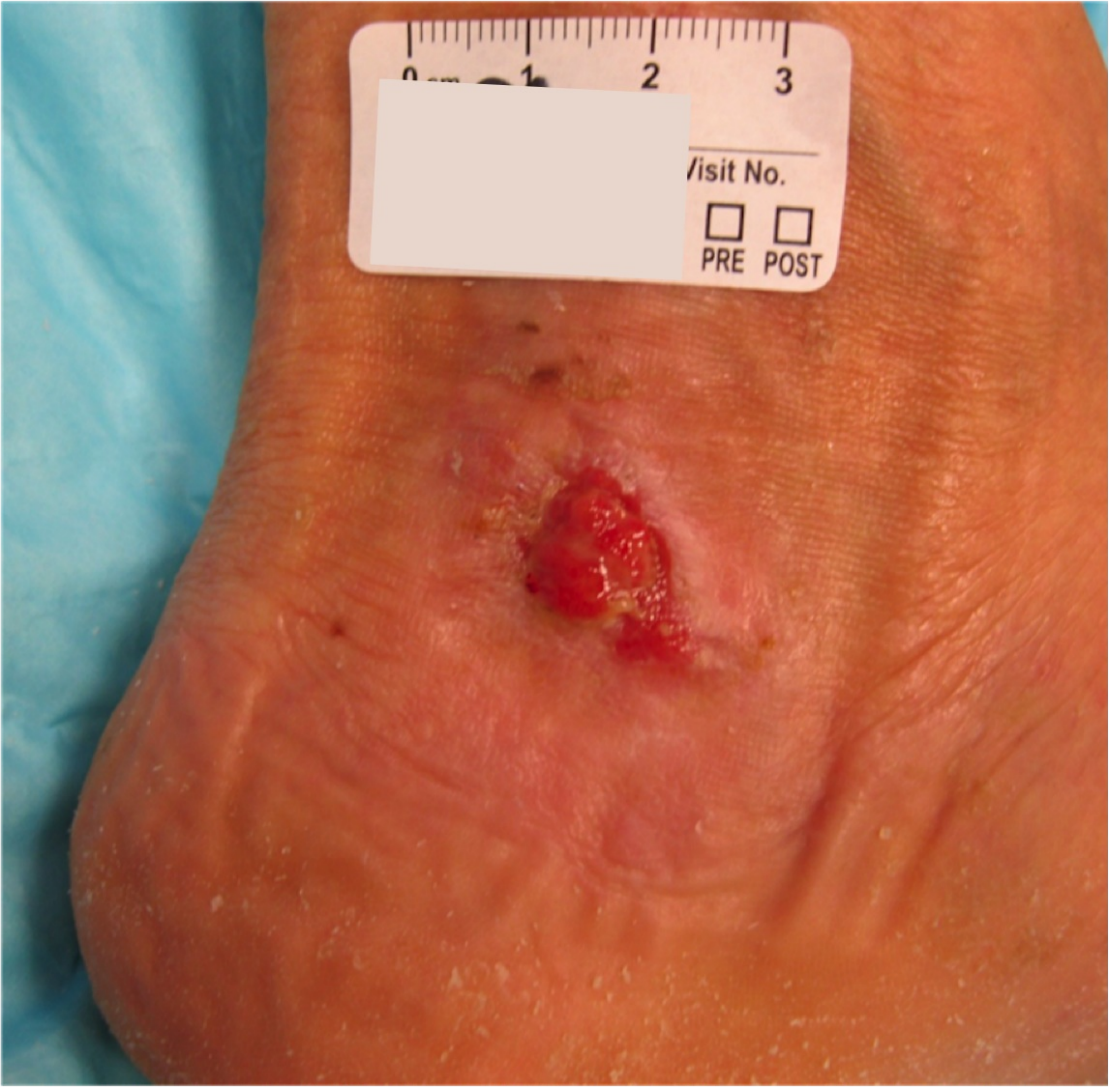
Case Three - Day 35

**Figure 11:**
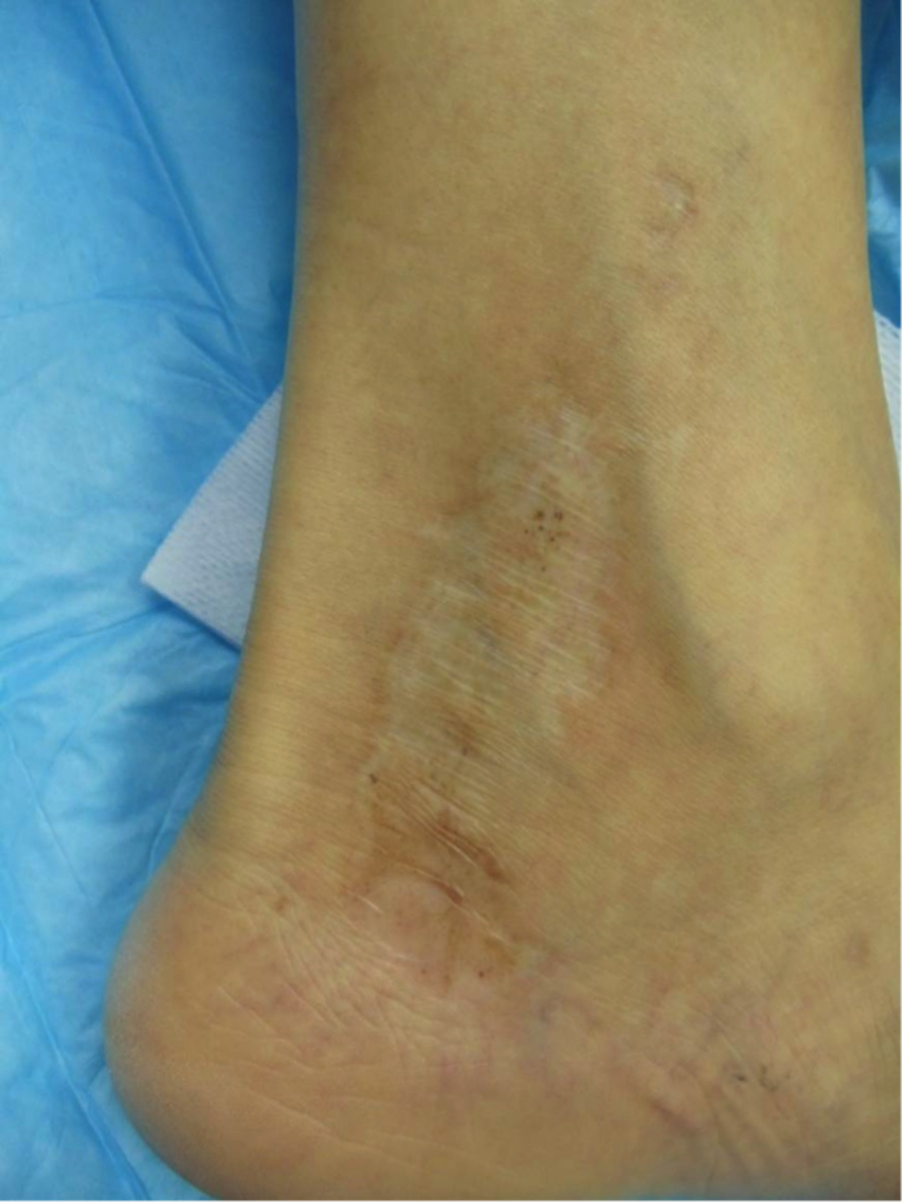
Case Three - Month Nine

The patient is a 28-year-old female with sickle cell disease presenting with a recurrent non-healing wound on the left medial leg for one year. Past treatments included aspirin, pentoxifylline, hydroxyurea, and regular blood transfusions, in addition to wound care therapy with compression therapy. Epidermal harvesting was performed using the CELLUTOME Epidermal Harvesting System from intact skin on the right thigh and transferred to the recipient site on the left medial leg via transparent film dressing. By week five, the patient had significant reduction in wound size and ultimately had closure at nine months (Figures 9-11).

Case Four

**Figure 12:**
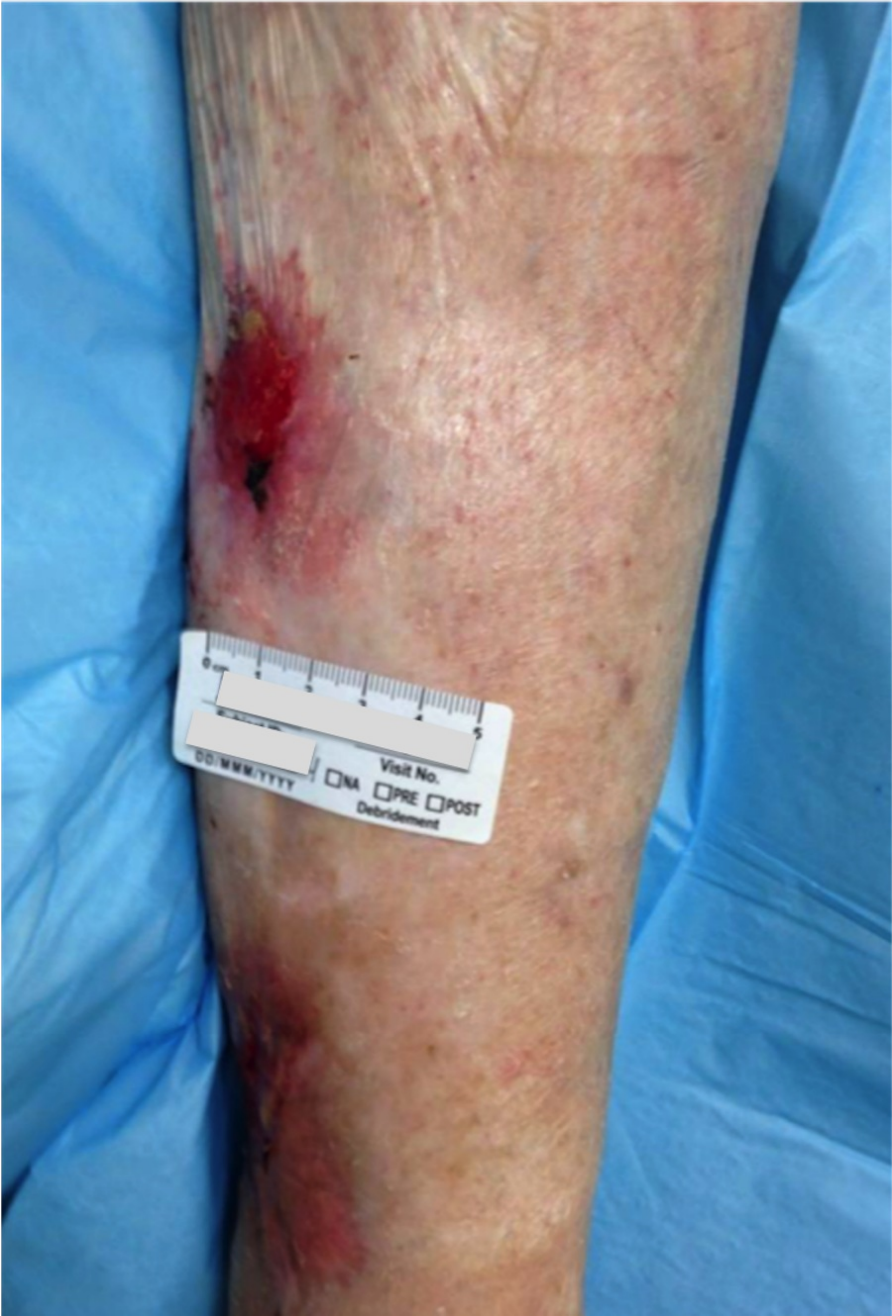
Case Four - Day Zero

**Figure 13:**
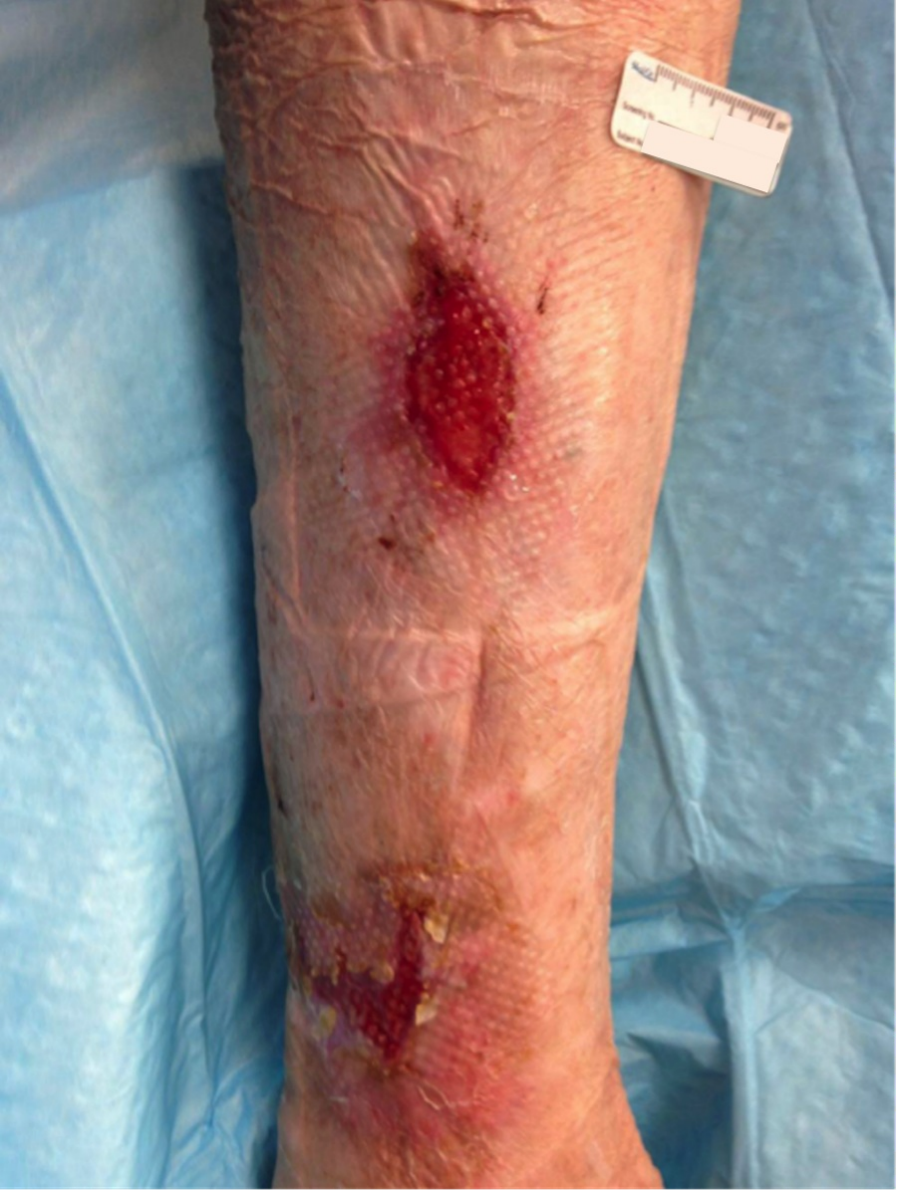
Case Four - Day Zero

**Figure 14:**
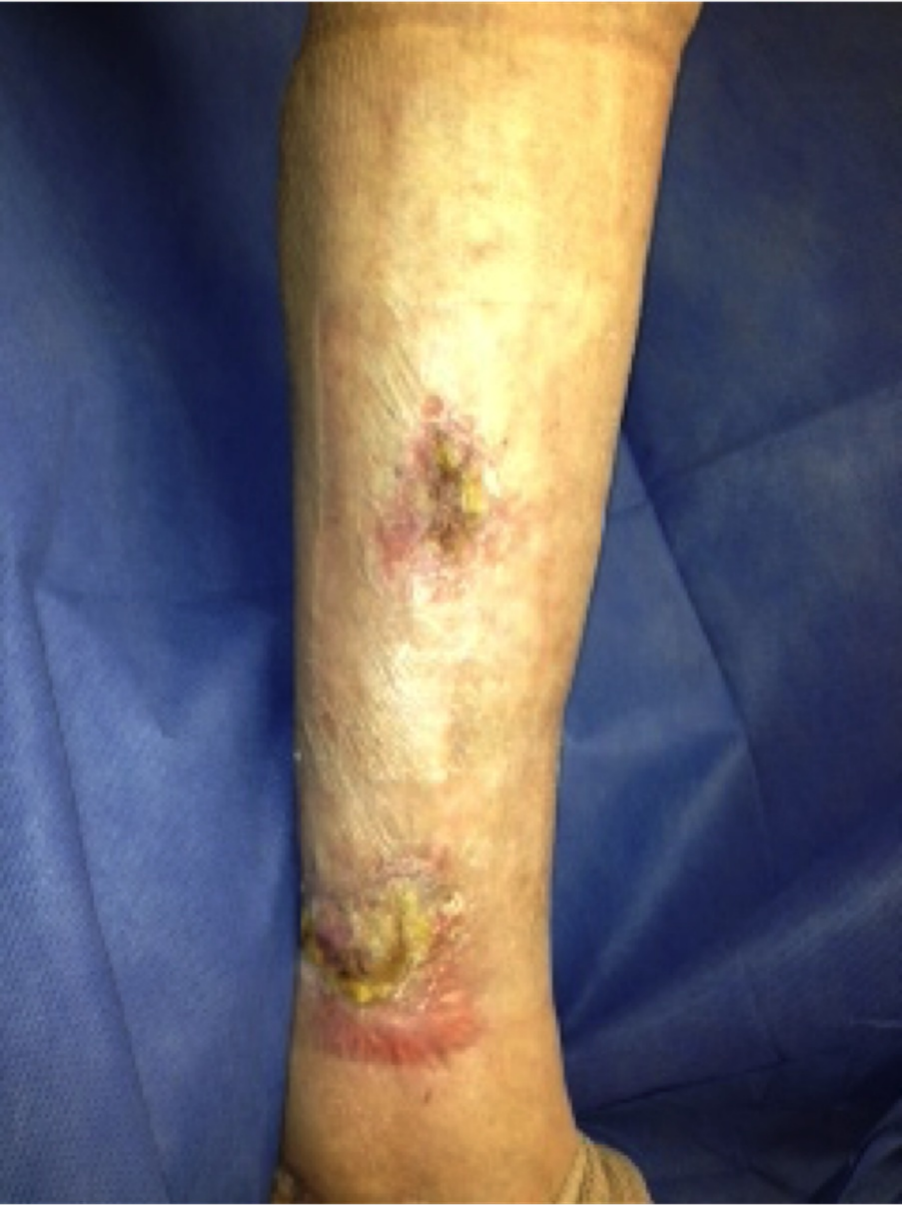
Case Four - Week 10

The patient is a 90-year-old male with venous insufficiency status post excision of a squamous cell carcinoma of the right lateral leg who developed a non-healing wound on the right lower extremity. His past treatments included compression and bilayered living skin equivalent therapy. Epidermal harvesting was performed using the CELLUTOME Epidermal Harvesting System from intact skin on the right thigh and transferred to the recipient site on the right lateral leg via transparent film dressing. The patient ultimately achieved wound closure at week 10 (Figures 12-14).

Case Five

**Figure 15:**
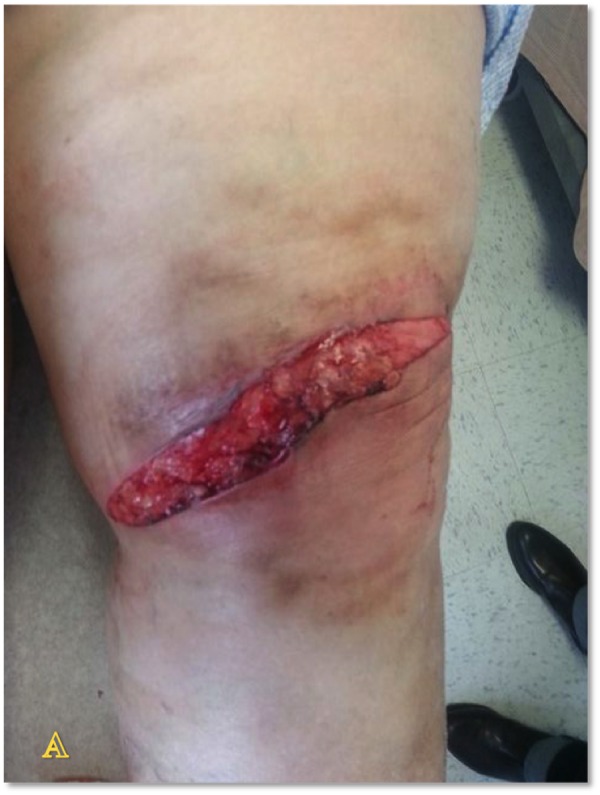
Case Five - Day Zero

**Figure 16:**
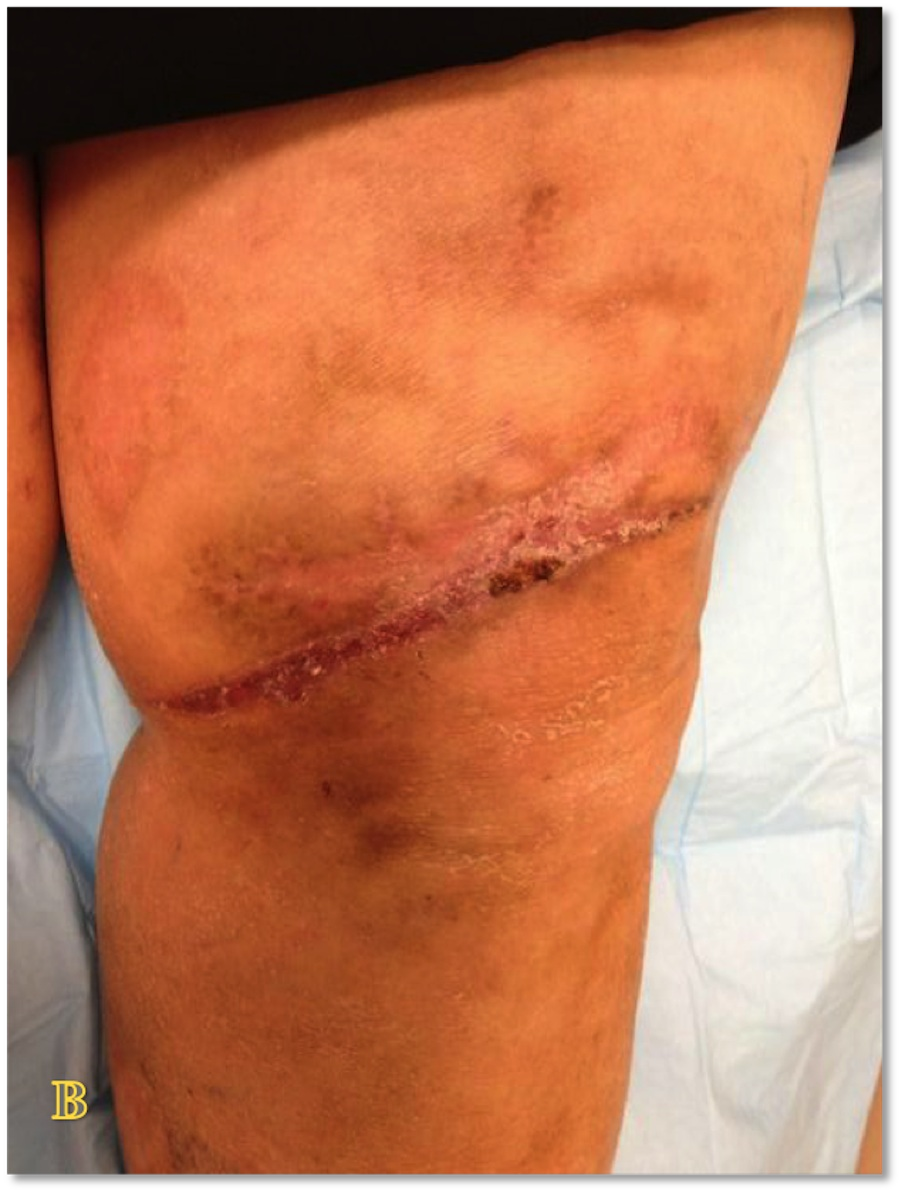
Case Five - Day 35

The patient is a 75-year-old female who developed a traumatic wound of the left distal thigh status post fall. Epidermal harvesting was performed using the CELLUTOME Epidermal Harvesting System from intact skin on the right thigh and transferred to the recipient site on the left distal thigh via transparent film dressing. At day 35, the patient achieved complete wound closure (Figures 15-16).

A review of the cases is outlined in Table 1.

**Table 1:** Table 1 Review of Epidermal Grafting Cases

Patient ID	Underlying Condition	Previous Treatment	Follow-up
Case One: 45-year-old male	Venous leg ulcer Pyoderma gangrenosum	Bilayered living skin equivalent Systemic steroids	Wound closure at day 60
Case Two: 78-year-old female	Venous leg ulcer	Bilayered living skin equivalent Small intestine submucosa Compression therapy	Significant reduction in size and depth at day 20
Case Three: 28-year-old female	Sickle cell ulcer	Aspirin Pentoxifylline Hydroxyurea Compression therapy	Significant wound size reduction at day 35; complete wound closure at nine months
Case Four: 90-year-old male	Post-surgical wound Venous insufficiency	Bilayered living skin equivalent Compression	Wound closure at day 70
Case Five: 75-year-old female	Post-traumatic wound	N/A	Wound closure at day 35

### Limited donor site morbidity

**Figure 17:**
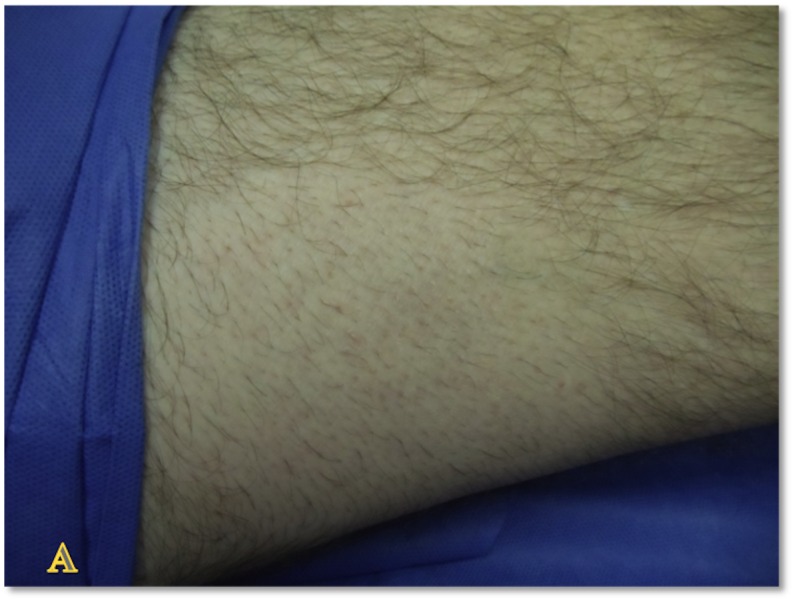
Epidermal Grafting Donor Site - Day Eight

**Figure 18:**
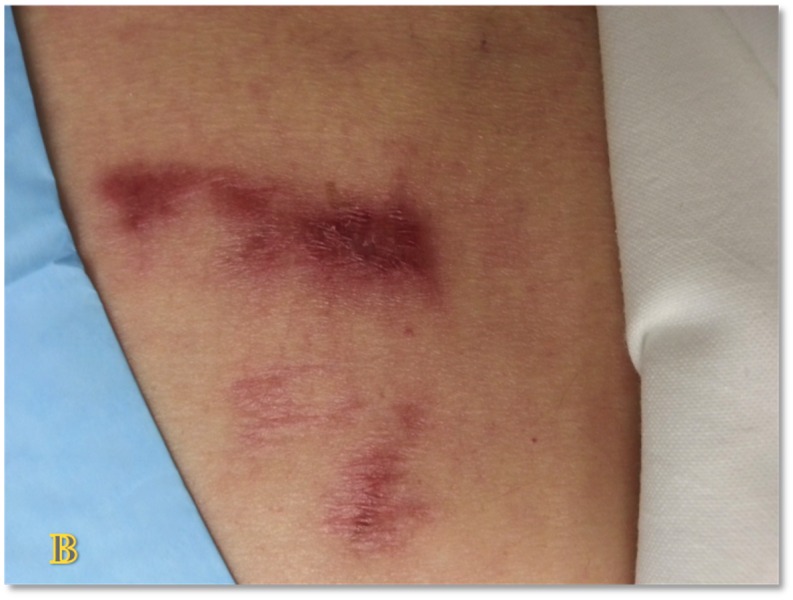
Punch Grafting Donor Site - Day 35

All patients had limited donor site morbidity with scarless healing and minimal discomfort with the procedure. When donor site healing using CELLUTOME was compared to punch graft donor sites, there was complete scarless healing of CELLUTOME donor site at day eight and residual scarring at punch graft donor site at day 35 (Figures 17-18).

## Discussion

Epidermal grafting represents an effective therapy for treatment of chronic wounds and, in our practice, was successful in healing several chronic lower extremity ulcers of various etiologies.

The exact mechanism of action of epidermal grafts is unknown. Typically, skin grafts take through three phases. The first, the plasmatic imbibition phase, is when the transplanted tissue absorbs wound fluid and gains up to 40% weight during the first 24 hours [3]. The second phase is the inoscularity phase, where anastomoses between donor and recipient skin are formed, and occurs two to three days after grafting [3]. Finally, the third phase is the revascularization phase, where there is vascular proliferation within both the donor and recipient tissues [3].

A new phase proposed to be important in epidermal grafting is called the keratinocyte activation phase [3]. After engraftment, the keratinocytes are “activated,” become proliferative, and express cell-matrix adhesion proteins such as beta 1 integrin. In a study by Yamaguchi et al., porcine wounds were treated with either epidermal grafts, STSG, or FTSG [3]. All grafts took well, and after engraftment, immunohistochemical analysis was performed for Ki67, a marker of cell proliferation, and beta 1 integrin. Beta 1 integrin has been shown to be a marker of cell-matrix adhesion and is upregulated at the edge of a wound healing site [20]. They found an upregulation of Ki67 and beta 1 integrin in epidermal graft- and STSG-treated wounds, as compared to low expression in FTSG [21]. As well, the expression of beta 1 integrin was found in more layers for wounds treated with epidermal grafts as compared to STSG. These results support that the activation of keratinocytes may be mediated by the interaction between epidermis and dermis, leading to production of important growth factors in wound healing, such as transforming growth factor-beta 1 (TGF-beta1) and PDGF [21].

The importance of an epithelial-mesenchymal interaction in wound healing between the grafted epidermis and recipient dermis has additionally been demonstrated in the treatment of palmoplantar wounds [22]. Palmoplantar skin differs from other body sites in both clinical attributes and histologic appearance. Palmoplantar skin is more resilient, responds differently to mechanical stress, and has differential keratin protein expression with high levels of keratin 9, which is considered a palmoplantar specific keratin [22]. Wounds on palmoplantar sites present a challenge as treatments with acceptable cosmetic outcomes are limited. Skin grafts performed from the medial pedal area, with palmoplantar skin, yield cosmetically agreeable results but have limited tissue supply. On the other hand, grafts applied from nonpalmoplantar sites result in poor cosmetic outcomes with hyperpigmentation, hyperkeratosis, and occasional hair growth in glabrous sites [22]. Additionally, grafts from these sites tend to be less durable and more at risk for erosions and/or ulcerations. Therefore, the ideal graft for these sites would be one with similar features to palmoplantar epidermis, and previous studies have shown that culture of palmoplantar fibroblasts with nonpalmoplantar keratinocytes resulted in expression of keratin 9 [23]. Therefore, it was postulated that the dermal cells can interact with the epidermal cells in such a way as to transform their protein expression to behave more like the recipient site.

In further evaluation of this observation, Yamaguchi et al. treated palmoplantar wounds with epidermal sheets and STSG, both derived from nonpalmoplantar sites [22]. Whereas the traditional STSG retained its nonpalmoplantar phenotype with hyperpigmentation and hyperkeratosis, the epidermal graft demonstrated adoption of palmoplantar phenotype with hypopigmentation [22]. This was supported on histology, where the areas treated with STSG showed acanthosis and elongation of saw-tooth rete ridges, whereas those treated with epidermal graft showed thick stratum corneum and acanthosis similar to normal palmoplantar epidermis. As well, immunohistochemistry was performed for keratin 9, which was negative in the epidermis of wounds treated with STSG and positive in wounds treated with epidermal grafting with similar distribution as normal palmoplantar epidermis [22]. This highlights that direct interaction between epidermis and dermis was necessary for this epithelial-mesenchymal interaction, leading to transformation of the donor site to be more consistent with the recipient site.

Keratinocyte activation and epithelial-mesenchymal interaction likely contribute to epidermal grafting success in part by production of appropriate growth factors and pro-healing cytokines. In line with cell-based skin therapies, which lead to wound closure not through engraftment but rather by stimulating healing, this could be, in part, the mechanism of epidermal grafts. This is demonstrated by the “edge effect,” where a major effect of healing is through stimulation of epithelialization from the edge of the ulcer and is thought to be mediated by growth factors produced by grafted keratinocytes [24]. In a case series of five patients treated with epidermal micrografts, it was noticed that the micrografts did not appear to “take” but instead stimulated healing from the wound edges [14]. Also, in a study by Costanzo et al. of 29 chronic, non-healing ulcers on the lower extremities treated with autologous epidermal grafting, they observed an increase in re-epithelialization from the wound edge, and ultimately, at 12 weeks, 89% of ulcers were healed [6].

## Conclusions

Epidermal grafting represents an effective treatment for chronic wounds. With the advent of a new harvesting system, epidermal grafting has become an easy, fast, and cost-effective treatment. Benefits over conventional STSG and FTSG include lower cost, decreased health care burden, reduced donor site morbidity, and improved patient satisfaction with donor site scar. It has also been shown to be effective in aiding healing of chronic wounds as demonstrated by several cases in our experience. The mechanism of action is not yet well understood, but it involves factors well beyond the placement of an occlusive dressing over a wound. These likely include production of growth factors and proinflammatory cytokines involved in wound healing, subsequent keratinocyte activation, and further epithelial-mesenchymal communication, resulting in both a cosmetically and functionally good outcome. Further research is needed to fully characterize the role, and additional uses in more patient settings should be explored to determine the full benefit of this exciting treatment modality.
